# Circulating stem cells, HIF-1, and SDF-1 in septic abdominal surgical patients: randomized controlled study protocol

**DOI:** 10.1186/s13063-018-2556-0

**Published:** 2018-03-12

**Authors:** Antonella Cotoia, Lucia Mirabella, Sabrina Altamura, Rachele Villani, Flavia Marchese, Giuseppe Ferrara, Karim Mariano, Tullo Livio, Gilda Cinnella

**Affiliations:** Department of Anesthesia, Intensive Care and Pain Therapy, University of Foggia, University Hospital Foggia, Foggia, Italy

**Keywords:** Stem cells, Major abdominal surgery, Sepsis, Septic shock, Stromal cell-derived factor 1a, Hypoxia inducible factor 1

## Abstract

**Background:**

Sepsis caused by complicated intra-abdominal infection is associated with high mortality. Loss of endothelial barrier integrity, inflammation, and impaired cellular oxygen have been shown to be primary contributors to sepsis. To date, little is known regarding the pathway for the mobilization of endothelial progenitor cells (EPCs) from the bone marrow in sepsis whereas stromal-cell-derived factor 1a (SDF-1a) and hypoxia inducible factor 1 (HIF-1) seem to have a role in the EPC response to hypoxic microenvironments.

The aims of the study are: (a) to determine the time course of the levels of circulating EPCs (CD133/CD34), SDF-1a, and HIF-1 in septic patients undergoing major abdominal surgery (group S), (b) to investigate the relationship between CD133/CD34, HIF-1, and SDF-1a, and (c) to investigate the relationship of these factors with the outcome of group S patients treated with standard conventional therapy alone (CT) or with the addition of extracorporeal hemoperfusion therapy (HCT).

**Methods/design:**

Patients undergoing major abdominal surgery will be allocated into groups: postoperative non-septic patients in an emergency surgical ward (group C) and postoperative septic patients in an intensive care unit (group S). The latter will be randomized to receive CT alone (S1) or with HCT (S2). Healthy volunteers (group H) will be recruited at University Hospital Foggia.

Peripheral blood (PB) samples will be collected preoperatively (T0), at 24 h (T1), 72 h (T2), 7 (T3), and 10 (T4) postoperative days in groups S and C, and at T0 in group H. The CD34/133 cells and HIF-1 counts will be determined by flow cytometer analysis. The concentration of SDF-1a in plasma will be calculated by enzyme-linked immunosorbent assay analysis (ELISA).

**Discussion:**

This prospective randomized clinical trial is designed to investigate circulating stem cells, levels of HIF-1 and SDF-1a, and their interrelationship in septic postoperative abdominal surgical patients treated with CT alone or with HCT. The rationale is that an integrated understanding of the role of hypoxia-related factors and EPCs in PB of septic patients could indicate which molecular processes need to be regulated to recover the innate immunity homeostasis. Understanding the function of EPCs in sepsis may provide innovative diagnostic and therapeutic approaches to improve the prognosis of this syndrome.

**Trial registration:**

ClinicalTrials.gov: NCT02589535. Registered on 28 October 2015.

**Electronic supplementary material:**

The online version of this article (10.1186/s13063-018-2556-0) contains supplementary material, which is available to authorized users.

## Background

Complicated intra-abdominal infection is a frequent cause of sepsis and septic shock in intensive care unit (ICU) patients [1, 2]. Sepsis caused by intra-abdominal infection is associated with higher mortality ranging on average 30% to 60% because of (a) the broad spectrum of infection and disease severity, (b) the crucial role of surgery in the management of intra-abdominal infection, and (c) the ongoing emergence of multidrug resistance in pathogenic organisms [3–6].

Evidence supports that the pathophysiology of sepsis moves from an initiating early/acute hyperinflammatory phase to a late/chronic hypoinflammatory and immunosuppressive phase [7]. More recently, loss of endothelial barrier integrity, inflammation, and impaired cellular oxygen have been shown to be primary contributors to sepsis-related organ dysfunction [8]. Endothelial progenitor cells (EPCs), a specific subtype of hematopoietic stem cell, are mobilized into the peripheral circulation under various pathologic conditions that are associated with vascular injury [9–14]. Mutunga et al. observed an increase of circulating endothelial cells during sepsis and concluded that endothelial damage occurs [9]. However, the quantification of EPCs in septic patients has had controversial results and Cribbs et al. demonstrated that the increased levels of EPCs were inversely associated with organ dysfunction [15]. To date, little is known regarding the relationship between EPC and organ dysfunction in septic postoperative abdominal surgical patients.

In addition, the pathway for the mobilization of EPCs from the bone marrow (BM) in sepsis or septic shock is still unknown. Emerging evidence suggests that stromal-cell-derived factor 1a (SDF-1a) mediates the migration of hematopoietic stem cells from BM to peripheral blood (PB), while hypoxia inducible factor 1 (HIF-1), which regulates SDF-1a expression, is the central mediator of the cellular response to hypoxic microenvironments [16].

### Aims of the study

Primary objective: To determine the time course of the levels of circulating EPCs (CD133/CD34), SDF-1a, and HIF-1 in septic patients undergoing major abdominal surgery.

Secondary objective 1: To investigate the relationship between CD133/CD34, HIF-1, and SDF-1a.

Secondary objective 2: To investigate the relationship between CD133/CD34, HIF-1, SDF-1a, and the outcome of sepsis and septic shock patients treated with standard conventional therapy alone (CT) or with the addition of extracorporeal hemoperfusion therapy (HCT).

In this trial, we hypothesize that levels of CD133/CD34, HIF-1, and SDF-1a will increase in septic surgical patients as a consequence of impaired tissue perfusion and cellular hypoxia. Our hypothesis is that the stimulation of hypoxia-related factors, like SDF-1a and HIF-1, could be the primary step in the stimulation of BM stem cells. Furthermore, we assume that surviving septic patients will show higher levels of EPCs, HIF-1, and SDF-1a.

## Methods/design

### Recruitment

This prospective observational clinical trial is designed to target postoperative patients undergoing major abdominal surgery and healthy volunteers at University Hospital Foggia. Participants who meet pre-specified eligibility criteria (Table 1) and provide written informed consent will be enrolled in the study.

**Table 1:** Eligibility criteria

Inclusion criteria	Exclusion criteria
Caucasian Over 18 years of age	When it is impossible to collect blood samples (organizational reasons or because of emergencies regarding the health of the patient)
	Pregnant patients
	Organ transplantation
	Palliative care
	Metastatic cancer patients

Detailed information on the benefits and risks of taking a blood sample will be provided to study candidates. Human blood samples and therapies will be acquired in accordance with institutional guidelines. All patients will be managed by applying standard protocols, following current guidelines and recommendations. The diagnosis of sepsis and septic shock will be made according to the new definitions [17].

This study is to be performed in accordance with the *World Medical Association Declaration of Helsinki: Ethical principles for medical research involving human subjects* (WMA 20 2013) [18], the *International Ethical Guidelines for Biomedical Research involving Human Subjects* (CIOMS 2002) [19], and the *ICH Harmonised Tripartite Guideline: Guideline for good clinical practice* (ICH 1996) [20].

The study has been approved by the local research ethics committee (Comitato Etico of Ospedali Riuniti, Foggia, Italy, 69/CE/2015). The trial has been registered with ClinicalTrials.gov (NCT02589535). We adhered to the SPIRIT statement (Additional file 1).

### Study design

Each day, the anesthesiologist in the operating room and the on-call anesthesiologist will alert the principal investigator to potential eligible patients. Participants included in the trial will be divided into two groups:

- Group C: Postoperative non-septic patients in an emergency surgical ward (control group)
- Group S: Postoperative septic shock patients in an ICU

**Fig. 1:**
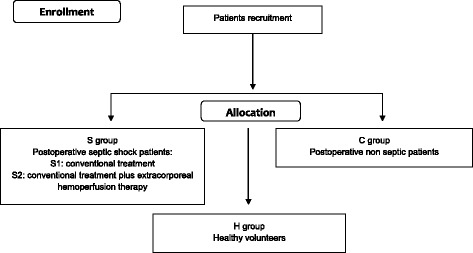
Work flow for the study. After recruitment, the participants will be allocated into one of two groups: postoperative septic patients in an intensive care unit (group S) or postoperative non-septic patients in an emergency surgical ward (group C). Healthy volunteers (group H) will be recruited from among staff members of University Hospital Foggia

**Fig. 2:**
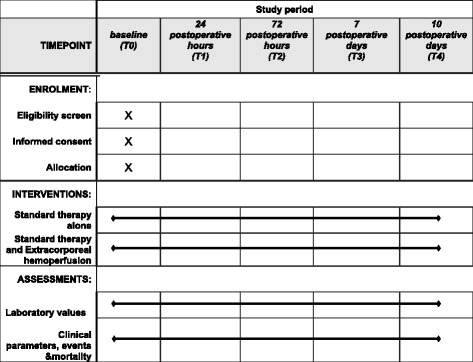
Schedule of enrolment, interventions, and assessments

Using a computer-generated sequence of numbers, patients in group S will be randomly assigned to receive either conventional therapy for septic shock alone (CT) as per the Surviving Sepsis Campaign guidelines (S1) or standard therapy plus extracorporeal hemoperfusion therapy (HCT) with polymyxin B-immobilized polystyrene-derived fibers to remove endotoxins from the blood (S2) [21]. Healthy volunteers (group H) will be recruited from among staff members of the University Hospital Foggia (Figs. 1 and 2).

### Blood sampling

PB samples will be collected in sterile evacuated blood collection tubes with anticoagulant ethylenediaminetetraacetic acid (EDTA; 3 ml) at the following time points:

- preoperatively (T0), at 24 h (T1), 72 h (T2), 7 (T3), and 10 (T4) postoperative days in groups S and C
- T0 in group H

The absolute numbers of leukocytes and lymphocytes in PB will be determined at the same time with an automatic cell counter (Cell-Dyn 3500, Abbott Diagnostics, Santa Clara, CA, USA). Peripheral whole blood samples (100 μl) will be used for fluorescence-activated cell sorting (FACS) analysis, and the plasma samples will be collected and stored at −80 °C.

Biochemical tests will be determined daily, according to the routine procedure in the surgical ward and ICU, and performed in the laboratories at University Hospital Foggia, according to the institutional guidelines.

Demographic, anamnestic, clinical, and laboratory data will be recorded.

### Flow cytometry

The samples of whole blood (100 μl) will be stained within 1 h from taking the blood sample. To analyze the viability of the cells, they will be stained with 7-aminoactinomycin D (7-AAD), which will bind to the DNA of cells that are undergoing or have undergone apoptosis (Stem Kit™ Beckman Coulter, Brea, CA, USA). The cell surface staining will be performed by adding the following fluorochrome-labeled monoclonal antibody reagents: fluorescein isothiocyanate (FITC)-conjugated anti-CD34 (Beckman Coulter, Brea, CA, USA) and phycoerythrin (PE)-conjugated anti-CD133 (Miltenyi Biotech, USA). Staining will be performed according to the manufacturer’s guidelines.

Cells will be fixed and permeabilized using the BD Intrasure kit (Becton Dickinson, Franklin Lakes, NJ, USA) and intracellular staining of HIF-1 will be performed using HIF-1 alpha (Alexa Fluor® 647 anti-human HIF-1α Antibody, BioLegend Inc., CA, USA) following the manufacturer’s instructions. The samples will be resuspended in phosphate-buffered saline and analyzed with a FACS Caliber flow cytometer (Becton Dickinson, Franklin Lakes, NJ, USA) and FCS3 software. Cell will be acquired with a four-parameter flow cytometry method (CD34 FITC/CD133 PE/CD45 PerCP/ HIF-1 ACP staining, side, and forward angle light scatter). The flow cytometer will be calibrated every 24 h and quality controls performed according to the UK National External Quality Assessment Service protocol. The number of CD34/CD133/HIF-1 cells in PB will be calculated as the count of absolute lymphocytes times the percentage (%) of gated CD34/CD133 positive cells, and expressed as absolute number of cells per 1 μl PB.

### Enzyme-linked immunosorbent assay

The plasma concentration of SDF-1a will be calculated by the enzyme-linked immunosorbent assay kit (SDF-1a human ELISA kit, Abcam, Italy) according to the manufacturer’s instructions.

### Statistical analysis

The power analysis suggested that a sample size of 30 patients per group is required to detect a higher percentage of total circulating EPCs (CD133+/CD34+ cells as a percentage of all myelomonocytic cells) in septic patients vs ICU controls (assuming α = .05 and power = .95) [22]. A sample size of three patients per group is required to detect higher SDF-1 levels in the septic vs control groups (assuming α = .05 and power = .95) [10]. This number is increased to 33 per group to allow for a 20% drop-out rate.

The normality of the distribution will be assessed by the Shapiro–Wilkinson test. If the data are normally distributed, they will be expressed as mean (± standard deviation). The data will be analyzed using the repeated measurements analysis of variance. Differences between the groups at each time point will be examined post hoc using an independent sample *t* test. A paired sample *t* test will be used to detect changes within the groups. The level of statistical significance was chosen as *P* < .05. The statistical analysis will be performed with the Statistical Package for the Social Sciences (SPSS Inc., Chicago, IL) version 15.0 for Windows.

## Discussion

The proposed study is a prospective observational clinical trial designed to investigate in septic postoperative abdominal surgical patients: (a) levels of circulating stem cells, HIF-1, and SDF-1a; (b) the interrelationship among stem cells, HIF-1, and SDF-1a; and (c) the correlation of those factors with outcomes for septic postoperative abdominal surgical patients treated with CT alone or with HCT. The rationale is that a deep and integrated molecular understanding of sepsis at the system level will indicate which molecular processes need to be regulated to recover the innate immunity homeostasis.

A potential limitation is that blood samples will be not collected in those patients operated on as an emergency over night or when the laboratory is closed, since the HIF-1 analysis requires immediate collection-to-storage times of fresh blood samples [23].

Understanding the function of EPCs in sepsis and sepsis-related organ dysfunction may provide innovative diagnostic and therapeutic approaches for this devastating syndrome.

### Trial status

Patient recruitment is currently being undertaken.

## Additional file

Additional file 1: SPIRIT checklist (DOC 120 kb)

## Abbreviations

BM: Bone marrow
CT: Conventional therapy alone
EPC: Endothelial progenitor cell
FACS: Fluorescence-activated cell sorting
HCT: Conventional therapy plus extracorporeal hemoperfusion therapy
HIF-1: Hypoxia inducible factor 1
ICU: Intensive care unit
PB: Peripheral blood
SDF-1a: Stromal-cell-derived factor 1a
